# Salophen

**Published:** 1893-05-27

**Authors:** 


### NEW DRUGS AND PREPARATIONS.

[We should be glad to receive, at our office, 428, Strand, London, W.C., from the manufacturers, specimens of all new preparations and drugs which may be brought out from time to time.]

### SALOPHEN.

Our personal experience of this drug fully accords with the evidence that has recently been brought forward to show that in salophen we have a compound of salicylic acid which possesses many advantages over the simple acid or its sodium salt. Its chemical name, acetyl-para-amydo-salol, expresses its composition. It is stable, and keeps well. In appearance it forms a mass of white crystalline scales, which are almost insoluble in cold water, moderately soluble in hot, and much more in alcohol or ether. It is stated by different authorities to contain 51 or more probably 50·9 per cent., of salicylic acid. In general action it is closely allied to salol, but is to be preferred for one or two reasons. In the human body it splits up into salicylic acid and acetyl-para-amydo-phenol. The latter, unlike ordinary phenol, is not a toxic substance, and even this decomposition only takes place in the alkaline medium of the small intestine, so that in the stomach we have merely the unirritating salophen itself.

Siebel believes that in all ordinary doses it is harmless, as well as odourless and tasteless; at least it is far less poisonous than salol. Sixty to ninety grains a day are well borne by patients. In rabbits it was found that not less than 7·5 grammes per kilogramme of the body weight were required for a fatal dose. It may be said to have been introduced to public notice by the interesting report of the results obtained under its use by Dr. Paul Guttman, the energetic director of the Moabit Hospital, Berlin, December, 1891.

He found that in cases of acute rheumatism of recent standing, salophen produced rapid disappearance of pain, though without any appreciable effect on cardiac complications. In a few instances, however, it seemed to fail, as indeed, salicylic acid is well known to do in certain cases. Whether these intractable cases depend on some peculiarity of the rheumatic poison, some failure of elimination, or some abnormality of the body metabolism, or on the special state of the tissues by which the poison is shielded from the effects of the drug, is a problem which urgently demands careful investigation. Such an investigation might not only explain much that is now obscure as to the nature of the disease, but lead to more rational and uniformly successful treatment.

Fröhlich confirms Guttman's favourable report as to its action in acute rheumatism for rapid relief of pain and for its harmlessness. He employed it in thirty instances, and found it just as good as sodium salicylate or salol. It has, moreover, great advantages, since it s not hygroscopic, and is devoid of taste. Even when ong continued and in large doses it has practically none of the after effects which follow the use of salicylate or salol. He could discover no symptoms of collapse, no giddiness, noise in the ears, no sickness, vomiting, or loss of appetite from its use. In short, in none of his thirty cases was there any derangement of the digestion, and only in three were there temporary cerebral symptoms. However, it does not prevent heart complications, nor can it be relied on always for chronic articular rheumatism, though occasionally affording relief. It appears useless for reducing temperature in other diseases.

Dr. Flint has recently published six cases of acute rheumatism in which he employed it. He gave fifteen-grain doses every three hours, as a powder placed on the tongue. The temperature fell to the normal on the second or third day of its administration, and he noticed no cardiac or digestive disturbances after its use.

Dr. Oswald, of Giessen, in describing the cases he treated by it, thinks that while the severest attacks of acute rheumatism require salicylates, many do thoroughly well on the safer and more agreeable salophen. Long-continued cases may well be transferred to salophen treatment, which is better borne and less weakening. For neuralgias and minor pains salophen is most valuable. The amount of salicylic acid given off to the blood does not depend on the dose, for with larger doses an increasing percentage passes through the intestine unbroken up. Hence the absence of symptoms under salophen which are seen when excessive doses of salicylates are given. Dr. Oswald employed from 60 to 120 grains a day.

Dr. H. A. Hare gives the results he has obtained by its use for minor ailments closely allied to rheumatism, which were highly satisfactory. He points out that theoretically it is much less poisonous than salol from its composition. It succeeded when salol failed to give relief in a case of violent neuralgia of the sciatic in a female of 65, to whom it was administered in pill form. In another case of neuralgia, involving the brachial plexus of a man aged 45, rapid relief was afforded by doses of 10 to 20 grains three times a day, after various other drugs had been given in vain. Similar success attended its use in neuralgia of the testicle and cord, and in a case of violent headache, with hyperæsthetic areas in the scalp, and in vomiting. Dr. Hare acknowledges that his cases were too few to warrant a definite estimate of its value, but its uniform success leads him to continue its use. He had no opportunity of testing it in acute rheumatism, but its agreeableness, and the absence of any interference with the digestion, and of other unpleasant results when continued, speak strongly in its favour. It will be important to discover how far it can be relied on as an intestinal antiseptic. One curious effect of its administration is noticed by Drasche. Profuse perspirations are apt to follow its use in acute rheumatism. When these evaporate the skin is covered by a film of fine pointed crystals, especially on the face, the arms, and on the folds of the joints. This white shining dust is composed of crystals of the same form as those of salophen itself, but whether they are of the same composition remains to be proved. However, the deposit does not appear to have any pathological significance.

On the whole, then, both in acute rheumatism, and in some analogous affections, salophen would seem to be a safe and active remedy, possessing special recommendations for practical use.

